# Dutch cardiology residents and the COVID-19 pandemic: Every little thing counts in a crisis

**DOI:** 10.1007/s12471-020-01519-6

**Published:** 2020-11-03

**Authors:** W. R. Berger, V. Baggen, V. M. M. Vorselaars, A. C. van der Heijden, G. P. J. van Hout, G. F. L. Kapel, P. Woudstra, the Junior Board (Juniorkamer) of the Netherlands Society of Cardiology (NVVC)

**Affiliations:** 1 Department of Cardiology, Onze Lieve Vrouwe Gasthuis, Amsterdam, The Netherlands; 2 Department of Cardiology, Erasmus Medical Centre, Rotterdam, The Netherlands; 3 Department of Cardiology, St. Antonius Hospital, Nieuwegein, The Netherlands; 4 Department of Cardiology, Leiden University Medical Centre, Leiden, The Netherlands; 5 Department of Cardiology, University Medical Centre Utrecht, Utrecht, The Netherlands; 6 Department of Cardiology, Medisch Spectrum Twente, Enschede, The Netherlands; 7 Department of Cardiology, Heart Center, Amsterdam UMC, University of Amsterdam, Amsterdam Cardiovascular Sciences, Amsterdam, The Netherlands; 8 Department of Cardiology, Medisch Centrum Leeuwarden, Leeuwarden, The Netherlands

**Keywords:** COVID-19, Cardiology, Training

## Abstract

The COVID-19 pandemic has overwhelmed healthcare systems worldwide, and a large part of regular cardiology care came to a quick halt. A Dutch nationwide survey showed that 41% of cardiology residents suspended their training and worked at COVID-19 cohort units for up to 3 months. With tremendous flexibility, on-call schedules were altered and additional training was provided in order for residents to be directly available where needed most. These unprecedented times have taught them important lessons on crisis management. The momentum is used to incorporate novel tools for patient care. Moreover, their experience of pandemic and crisis management has provided future cardiologists with unique skills. This crisis will not be wasted; however, several challenges have to be overcome in the near future including, but not limited to, a second pandemic wave, a difficult labour market due to an economic recession, and limitations in educational opportunities.

The COVID-19 pandemic has hit cardiology patients hard, as they are susceptible to a severe course of their disease [1]. Due to the very rapid and unprecedented increase of COVID-19 patients, the regular cardiology care came to a quick halt. Cardiology residents all over the Netherlands were reallocated to COVID-19 cohort units. With tremendous flexibility, on-call schedules were altered and additional training was provided in order for residents to be directly available where needed most.

A questionnaire of the Junior Board (*Juniorkamer*) of the Netherlands Society of Cardiology (*NVVC*) showed that 41% of cardiology residents were involved in frontline COVID-19 care throughout the Netherlands (Fig. 1). The questionnaire was completed by 122 residents from 36 clinics in the Netherlands; residents at every stage of the 6‑year training programme from all 15 so-called ‘A-clinics’ (i.e. clinics which are leading the training programme) were included. They worked at COVID-19 cohort units for 1–3 months, while regular training programmes were suspended. When the pandemic hit hard, these young doctors felt a great responsibility to do whatever they could—within their competencies—and to do their share on the wards and intensive care units. A similar pattern was seen worldwide [2].

**Fig. 1:**
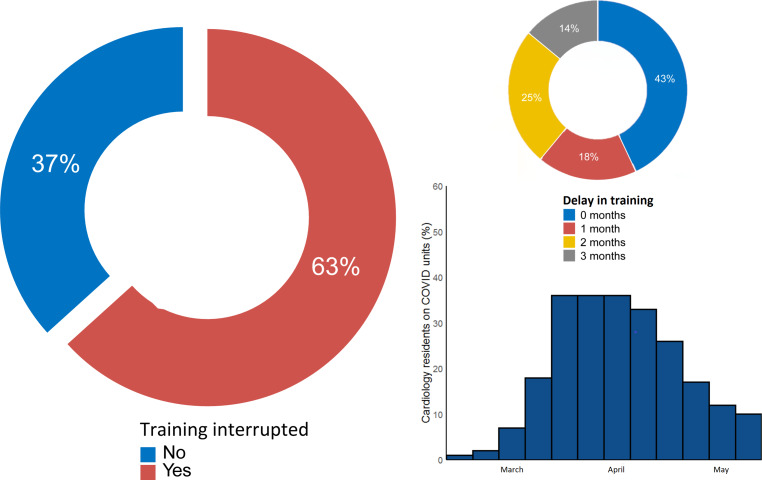
Results of nationwide survey among 122 Dutch cardiology residents during COVID-19 pandemic

The willingness of staff to enable reallocation of a large part of the residents proved to be of great support. After the first decline in the number of COVID-19 patients in Dutch hospitals, it is time to think about the lessons learned and to reshape the future.

## Lessons learned

The impact of this pandemic on regular healthcare could not have been predicted. The tremendous need of resources urged nurses, physicians and supportive staff to rethink processes of daily care in order to continue acute care, to prevent spread of the coronavirus and to limit the use of scarce protection gear. Residents were directly involved in crisis management. With their great day-to-day working experience in patient care, they helped to redefine the processes of emergency, clinical and outpatient care. While physicians are trained to be ready, the magnitude of this crisis could only be dealt with using real-life experience.

The COVID-19 crisis has shown the importance of teamwork in healthcare. Residents have shown flexibility in both the continuation of regular healthcare for the (acute) cardiac patient and dedicated care for COVID-19 patients. Moreover, the efforts of cardiologists who were involved in tasks that are normally performed by residents increased flexibility and warranted continuation of regular (acute) care. Once again, the healthcare system proves to be an efficient engine that depends on a great team effort of, but not limited to, technicians, nurses, facility services, security personnel, pharmacists and stretcher-bearers [3].

COVID-19 showed its many faces in the course of time. Residents are continuously implementing their observations in day-to-day care, together with new knowledge, which has been shared by the many publications on this topic [4]. They have followed crash courses in viral infections, epidemiology, advanced respiratory care, thrombosis, haemostasis, et cetera. Moreover, cardiology residents have proven to be essential in the often ad hoc created multidisciplinary teams of doctors given their advanced knowledge of haemodynamics and interpretation of side effects of medications (e.g. chloroquine) on cardiac conduction and function [5, 6].

eHealth solutions were readily made available to proof their value as an efficient alternative to face-to-face contact. Daily plenary teaching moments were replaced by on-demand virtual meetings. Cardiology-specific training, as provided by the Cardiovascular Teaching Institute (*CVOI*), underwent fast and rigorous innovations in online medical education. Worldwide eHealth and virtual leaning opportunities have gained an enormous momentum due to the circumstances, and we know they are here to stay [7, 8].

The necessary measures to prevent further spread also changed behaviour and manners in patient care. A hand on the heart or a ‘low bow’ has replaced the now old-fashioned handshake to welcome a patient. Family visits for admitted patients were limited to a bare minimum and communications were mainly made by phone or videophone. These included emotional and difficult conversations, such as end-of-life discussions.

The COVID-19 crisis improved our abilities as a doctor; it taught healthcare workers to be aware of their behaviour and to improve their communication skills (Tab. 1). The COVID-19 pandemic showed once again our humility toward nature and reinforced a skill that may sometimes be forgotten during medical training: compassion. At the same time, we experienced that everyday social interaction with colleagues is of great importance to cope emotionally with the heavy workload and the often grievous impressions this crisis has brought us.

**Table 1:** Lessons learned from COVID-19 healthcare crisis for cardiology residents

*Knowledge*
– Pandemic and disease control measures
– Development of novel disease characteristics and treatment protocols
– Respiratory care on COVID-19 cohort units and intensive care units
*Management*
– Crisis management structures
– Opportunities for and limitations of a healthcare system
– Multidisciplinary improvement of care
*Innovation*
– Implementation of eHealth solutions
– Implementation of virtual learning
*Communication and collaboration*
– Teamwork and compassion are cornerstones of healthcare system
– Alternative (virtual) patient and family communication
– Importance of well-organised aftercare, such as peer support

## Challenges in the near future

The experiences gained during the COVID-19 pandemic have taught the residents many lessons, even though almost half of the cardiology residents reported a delay in their cardiology training of 1–3 months (Fig. 1). To prevent gaps in training or knowledge, a personalised restructuring of the training programme is necessary for many cardiology residents. This new training scheme will be implemented in an era in which several important constraints to daily care resulting from social distancing are still valid. This could limit the exposure of residents to clinical cases and training procedures. However, we have to utilise the current circumstances to introduce new training methods, such as virtual reality education, distance learning or advanced teaching, to improve learning efficiency. Residents have to work together with their mentors in teaching hospitals and to keep being creative and flexible in order to create practical solutions.

The pandemic has an enormous economic impact, also on the Dutch healthcare system. We hope that the (financial) uncertainties that lie ahead do not hinder the future careers of cardiology residents. These future cardiologists, who conquered COVID-19 in the frontlines of healthcare with tremendous effort and flexibility, are well prepared for a great future in clinical care. The lessons they learned will be of great importance for a paradigm shift to a more pandemic-resistant society and a modern healthcare system with an accelerated introduction of eHealth solutions. Since we are in a second wave of coronavirus infections, we need to work together with all stakeholders to be prepared for the (near) future.
